# Identification of the oxidation stress-related gene signatures and functional verification of MINK1 in prostate cancer cells

**DOI:** 10.1371/journal.pone.0350334

**Published:** 2026-07-08

**Authors:** Shuai Liang, Shuhua Zhou, Yangshuo Tang, Moyan Xiao, Ke Ye

**Affiliations:** 1 Department of Pancreatic Surgery, Xiangya Hospital, Central South University, Changsha, Hunan, China; 2 Comprehensive Surgery, Xiangya Boai Rehabilitation Hospital, Changsha, Hunan, China; 3 Department of Ultrasound Medicine, Xiangya Hospital, Central South University, Changsha, Hunan, China; 4 Department of Hepatology, Xiangya Hospital, Central South University, Changsha, Hunan, China; Nantong University, CHINA

## Abstract

**Background:**

A major challenge facing prostate cancer (PCa) cells is oxidative stress, yet the precise role and underlying mechanisms remain inadequately elucidated. The study sought to investigate the association between oxidative stress and PCa prognosis, as well as to identify potential regulatory pathways involved.

**Methods:**

Oxidative stress-related genes and data were sourced from the Genecards, TCGA-PRAD, and GSE16560 databases. A risk model was developed using machine learning, including random forest and LASSO analyses. Survival analysis, functional and immune infiltrate analysis, as well as immunotherapy analysis were performed. The expression of MINK1 was examined, and the effects of MINK1 silencing on cell biological activities (including proliferation, migration, and invasion) were investigated, alongside analyses of MINK1-related pathways.

**Results:**

Four genes (BCO1, MINK1, TAF1C, and MIS18BP1) were identified and utilized to develop an oxidative stress-risk score (OS-score). The high-OS-score predicted a poor prognosis, and OS-score was an independent prognostic factor for PCa. The OS-score demonstrated a positive correlation with CD8 + T cells, activated CD4 + T cells, and macrophages. Patients classified within the high-OS-score group were more effective in anti-PD-1 therapy (Nominal *P* = 0.001, Bonferroni corrected *P* = 0.011). A significant disparity was observed in the efficacy of immune checkpoint inhibitor treatment between the high- and low-OS-score groups (*P* = 1.1 × 10^−9^), with a higher proportion of responders in the high-OS-score group compared to non-responders. The key oxidative stress gene, MINK1, was experimentally validated and found to be highly expressed in PC-3 and DU145 cell lines. Silencing MINK1 resulted in decreased proliferation, migration, and invasion activity. Additionally, MINK1 knockdown induced G0/G1 phase arrest and inhibited nuclear translocation of NF-κB.

**Conclusion:**

In summary, oxidative stress is associated with a poor prognosis in PCa. Oxidative stress-related gene MINK1 may regulate the cell biological activity through cell cycle and NF-κB signaling pathway. This study may provide new clues for the identification and development of new markers for the diagnosis and prognosis of PCa patients.

## Introduction

Prostate cancer (PCa) ranks as the second most frequently diagnosed malignancy among men [1]. The incidence of PCa has persistently remained high [2], contributing to an escalating social burden [3]. PCa is a typically heterogeneous disease [4]. There are significant differences in clinical manife stations among patients, some of which are accompanied by metastatic invasive diseases, while others are inert diseases that progress slowly [5]. Immunotherapy has emerged as a promising treatment modality for various malignancies [6–11]. However, in the context of PCa, its application is still under investigation, and androgen deprivation therapy (ADT) continues to be the primary treatment approach during metastatic stages [12–14]. The long-term oncological outcomes for PCa patients vary considerably [15], underscoring the urgent need for novel biomarkers to inform the development of potential therapeutic strategies.

Oxidative stress results from the disruption of homeostasis due to the excessive accumulation of reactive oxygen species (ROS) [16–18]. ROS are predominantly generated through the electron transport chain in mitochondria, as well as in peroxisomes and the endoplasmic reticulum, where protein oxidation occurs [19]. Previous research indicates that oxidative stress is involved in the development of multiple cancers, including bladder cancer [20], ovarian cancer [21], gastric cancer [22], and PCa [23,24]. Chronic oxidative stress, which is associated with the progression of invasive and metastatic diseases, plays a critical role in meeting the heightened metabolic demands resulting from the rapid proliferation of cancer cells [19,25]. Concurrently, ROS and cellular oxidative stress are produced excessively when cells are subjected to physical environments, cancer chemotherapy and radiotherapy [26,27]. In PCa, the continuous evolution of androgen receptor signaling can dynamically regulate the NRF2 activity related to oxidative stress and ROS levels [28]. Androgen receptor-targeted therapy has been shown to induce oxidative stress in PCa [29]. Furthermore, oxidative stress can activate the androgen receptor signaling pathway to drive the castration resistance of PCa [30]. Maintaining the balance of oxidative stress levels within cells is crucial for cell viability. Our research focuses on oxidative stress in PCa.

Machine learning is increasingly important in predicting prognosis and developing new therapeutic strategies for targeted genes [31–34]. It has been extensively utilized to screen and identify characteristic genes in various cancers, including triple-negative breast cancer [35], low-grade glioma (LGG) [36], and colorectal cancer [37]. Here, we integrated oxidative stress-related genes and constructed an oxidative stress-related risk score (OS-score) via machine learning. The relationship between OS-score and gene mutation and immunotherapy was analyzed. The effects of OS-score on the prognosis of PCa patients were also determined from the Cancer Genome Atlas (TCGA) and Gene Expression Omnibus (GEO) databases. Our results will provide a research direction for predicting the prognosis and treatment of PCa.

## Materials and methods

### Data acquisition and pre-processing

**Fig 1:**
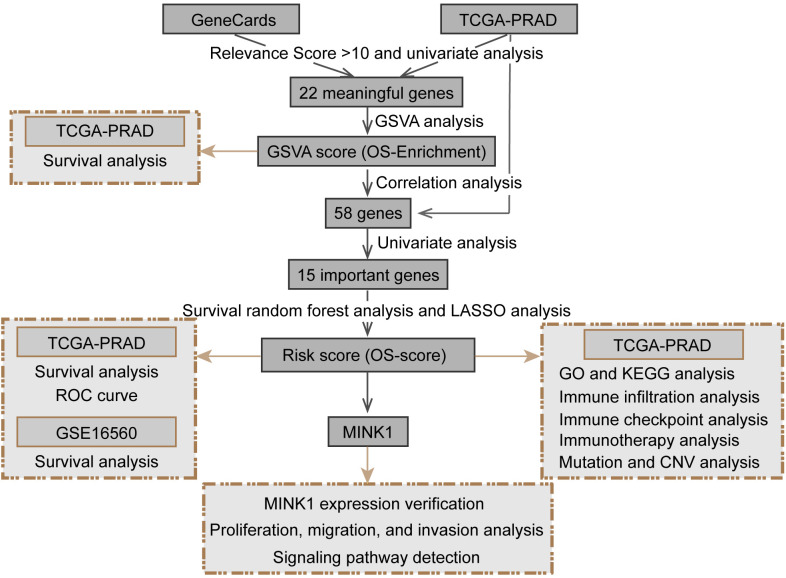
The flowchart of the study.

The TCGA-PRAD dataset was obtained from the UCSC Xena platform (https://xenabrowser.net/). GSE16560 (GPL5474), containing 281 cases, was downloaded from the GEO database (https://www.ncbi.nlm.nih.gov/gds/). Following the acquisition of annotation data, probes were mapped to their corresponding genes. In instances where multiple probes corresponded to a single gene, average expression values were computed. All data were normalized as described previously [38]. The workflow of this study is depicted in Fig 1.

### Construction of oxidative stress-related prognostic model

From Genecards, 9555 genes related to oxidative stress were downloaded. The relevance Score > 10 was applied as screening criteria to obtain the 422 genes. Next, the univariate analysis was executed and acquired 22 meaningful genes (*P* < 0.05). The Gene Set Variation Analysis (GSVA) score of meaningful genes was obtained using the GSVA analysis. The correlation analysis between the GSVA score and all genes were executed, with the correlation coefficient > 0.35 (all positive), *P* < 0.05 as the standard. The univariate analysis (*P* < 0.05) and survival random forest analysis (the relative importance > 0.3) were applied to analyze the GSVA score-related genes. Then, we performed a second-round screening using LASSO Cox regression to improve the model’s generalizability and eliminate high correlations among genes. By determining the optimal penalty parameter $\lambda$ via 10-fold cross-validation, the coefficient of one redundant gene (BAZ1A) was successfully shrunk to zero. As a result, four key genes (BCO1, MINK1, TAF1C, and MIS18BP1) with the strongest independent predictive value were ultimately selected to construct the Risk Score model. The risk score (OS-score) was composed of the gene expression and coefficient. Risk score = 4.1028 × BCO1 + 1.0111 × MINK1 + 0.4966 × TAF1C + 3.9616 × MIS18BP1.

### Functional and immune infiltrate analysis

Gene set enrichment analysis (GSEA) was conducted using the prognosis index via the R package clusterProfiler. According to four algorithms (ESTIMATE, MCPCounter [39], ssGSEA, and TIMER [40]), the immune infiltrating cells was estimated by “IOBR” package with prognosis index.

### Immunotherapy prediction

The Subclass Mapping (SubMap) algorithm [41] was employed to assess expression similarity between high- and low-OS score and patients undergoing anti-PD-1 and anti-CTLA-4 treatments. The Tumor Immune Dysfunction and Exclusion (TIDE) algorithm was utilized to predict patient responses to immunotherapy.

### Mutation and Copy number variation (CNV) analysis

Somatic mutations and CNV profiles were obtained from the TCGA dataset. The “maftools” package was utilized to delineate the mutational profiles of PCa patients stratified by high- and low-OS groups. CNV analysis was conducted for these groups using GISTIC 2.0.

### Cell Culture and transfection

RWPE-1, PC-3, and DU145 cells were cultured as described previously [38]. The human PCa cell line 22RV1 (Abiowell, Changsha, China) was cultured in RPMI 1640 medium supplemented with 10% fetal bovine serum and 1% penicillin–streptomycin. All cells were cultured in 5% CO_2_, at 37℃ humidified incubator.

The negative control (NC) siRNA and MINK1 siRNA-(1–3) were transfected into the cells with Lipofectamine™3000 (Invitrogen, Carlsbad, CA, USA). NC siRNA and MINK1 siRNAs were acquired from Sangon Biotech (Shanghai) Co., Ltd. The sequences are: MINK1 siRNA-1: sense- GCGGAUUAAGUUCCUGGUCAUTT, antisense- AUGACCAGGAACUUAAUCCGCTT; MINK1 siRNA-2: sense- GCUACUGAAGUUUCCCUUCAUTT, antisense- AUGAAGGGAAACUUCAGUAGCTT; MINK1 siRNA-3: sense- GAACCGUAACUGCAUCAUGAATT, antisense- UUCAUGAUGCAGUUACGGUUCTT; and NC siRNA: sense- UUCUCCGAACGUGUCACGUTT, antisense- ACGUGACACGUUCGG AGAATT.

### RNA extraction and quantitative reverse transcription (RT)-PCR analysis

Total RNA was extracted from the cells using the Trizol method. Reverse transcription was performed using the HiFiScript cDNA Synthesis Kit (CW2569, CWBIO, Beijing, China). RT-qPCR was conducted using the UltraSYBR Mixture (CW2601, CWBIO, Beijing, China). The relative mRNA levels of MINK1 were normalized and calculated via the 2^−ΔΔCt^. The primers are MINK1: Forward- CATGAATCCCGCTGACAAACCA, Reverse- GAGTTCTGCTGCTTGTTCATCC; BCO1: Forward-CCACAGTACCAGAGGGCAAG, Reverse-GGGATGGAGCAGAACACCTC; TAF1C: Forward-GAGGCCCATTTTCCAGACGA, Reverse-AACAAGGACCAAGCACCACA; MIS18BP1: Forward-GTGTTAGAGAACGTCGCGGA, Reverse-CAGAAACCTTGGATGAAGAGCC;

GAPDH: Forward- ACAGCCTCAAGATCATCAGC, Reverse- GGTCATGAGTCCTTCCACGAT.

### Western blot

Total protein was extracted using Radio Immunoprecipitation Assay (RIPA) buffer (AWB0136, Abiowell, Changsha, China). Nuclear and cytoplasmic proteins were isolated utilizing the NE-PER kit (Thermo Fisher Scientific, Waltham, USA). Protein quantification was conducted with the Bradford Protein Assay Kit (AWB0105b, Abiowell, Changsha, China). The protein samples underwent separation via 10% sodium dodecyl sulfate–polyacrylamide gel electrophoresis and were subsequently transferred to a nitrocellulose membrane. Membranes were blocked with 5% skim milk. Primary antibodies, including MINK1 (1:500, 13137–1-AP, proteintech, Chicago, USA), NK-κB (1:1000, PA5-17150, Invitrogen, Carlsbad, CA, USA), PCNA (1:1000, ab92552, abcam, Cambridge, UK), and β-actin (1:5000, 66009–1-Ig, proteintech, Chicago, USA), were incubated with the membranes. Following this, membranes were incubated with secondary antibodies. SuperECL Plus (AWB0005, abiowell, Changsha, China) and chemiluminescence imaging system were performed to visualize the protein levels. Total and cytoplasmic proteins were normalized to β-actin, while nuclear proteins were standardized relative to PCNA.

### Cell Counting Kit-8 (CCK-8) assay

The proliferative capability of PC-3 and DU145 cells was analyzed assessed using the Cell Counting Kit-8 assay (Dojindo, Japan) as described previously [42]. Cells were seeded onto 96-well plates (5 × 10^3^ cells/well). The proliferative activity was detected at 0 h, 12 h, 24 h, and 36 h.

### Plate clone formation assay

Plate clone assay was conducted to evaluate cell proliferation capacity. Cells were seeded onto 6-well plates at a density of 200 cells per well. After 2 weeks, cells were fixed using 4% paraformaldehyde and stained with 0.1% crystal violet. The resulting colonies were counted and photographed.

### Wound healing assay

A wound healing assay was performed using PC-3 and DU145 cell lines. PC-3 and DU145 cells were cultured in 6-well plates (5 × 10^5^ cells per well). PCa cells were more than 80% confluent and then were scratched with a 200 μl pipette tip. The wells were washed and loosely attached cells were removed. Photographs were taken at 0 h, 24 h, 48 h, and the healing percent was calculated by Image.

### Transwell assay

Invasion assays were operated in a Transwell chamber system. Transwell inserts were coated with 200 μg of Matrigel. PC-3 and DU145 cells were seeded into the upper chamber at a density of 2 × 10^5^ cells per well, while the lower chamber contained 500 μl of medium with 10% serum. After 48 h, the chambers were removed. Remaining cells were wiped off using cotton swabs. Cells were then fixed with 4% paraformaldehyde and stained with 0.1% crystal violet. The stained cells were observed under a light microscope and photographed.

### Apoptosis analysis

Apoptosis was assessed in accordance with the manufacturer’s protocol utilizing the Annexin V-APC/ Propidium iodide (PI) apoptosis kit (KGA1030, KeyGEN BioTECH, Nanjing, China). PC-3 and DU145 cell lines were subjected to washing and collection processes, followed by incubation of 5 × 10^5^ cells with 5 μl of Annexin V-APC and 5 μl of PI for 10 min. Apoptotic events were subsequently analyzed via flow cytometry.

### Cell cycle analysis

The cell cycle analysis was determined using PI (MB2920, Meilunstar, Dalian, China) as per the manufacturer’s guidelines, involving the addition of 150 μl of PI was added for staining at 4℃ for 30 min, after which the distribution of cells across the G0/G1, S, and G2/M phases was quantified using a flow cytometry.

### Statistical analysis

The Shapiro-Wilk normality test was employed to assess the normality of data distribution. For statistical analysis, normally distributed variables were evaluated using a two-tailed Student’s t-test, one-way ANOVA or two-way ANOVA. For the non-normally distributed variables, Wilcoxon Rank-Sum test or the Kruskal-Wallis test was applied. Data visualization was performed using the “ggplot2” package in R. The Kaplan-Meier method was employed to estimate the survival time of PCa patients and to generate Kaplan-Meier survival curves. Receiver operating characteristics (ROC) curve was generated using the R package “pROC”. *P* < 0.05 were regarded as significant statistical difference.

## Results

### Screening of oxidative stress-related genes in PCa

**Fig 2:**
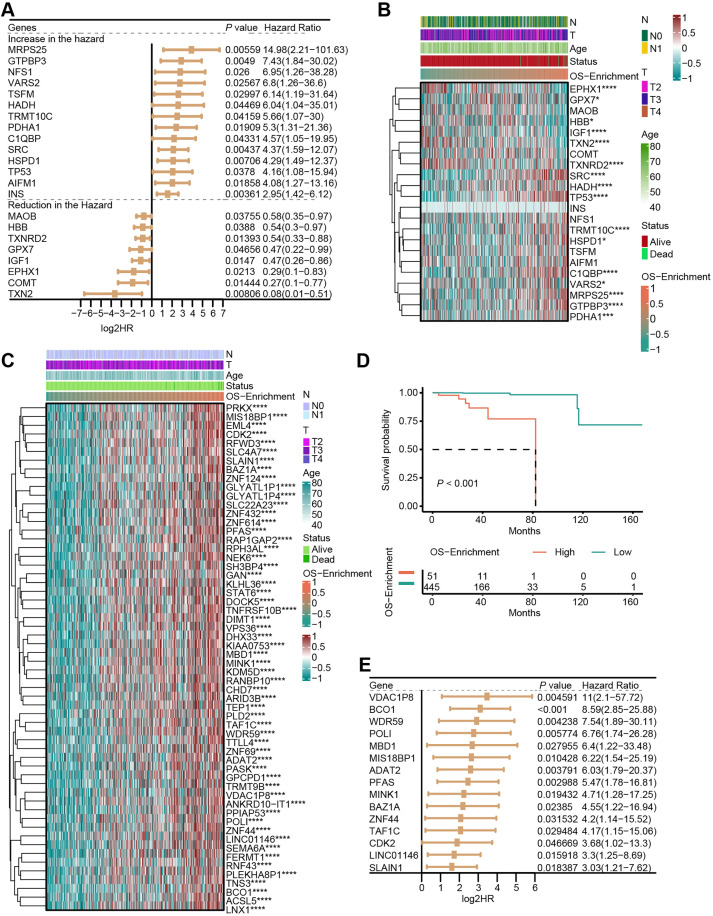
Screening oxidative stress-related genes in PCa. **(A)** Univariate analysis revealed 22 meaningful genes. **(B)** Heat map visualization of meaningful gene expression. **(C)** The correlation analysis between GSVA score and all genes was performed and obtained 58 related genes. **(D)** Survival analysis, based on the TCGA-PRAD dataset. **(E)** 15 related genes screened by univariate analysis.

We obtained oxidative stress-related genes from GeneCards and sourced 422 genes with a relevance score > 10. Subsequently, a univariate analysis was conducted, resulting in the identification of 22 meaningful genes (Fig 2A), including MRPS25, GTPBP3, NFS1, VARS2, TSFM, HADH, TRMT10C, PDHA1, C1QBP, SRC, HSPD1, TP53, AIFM1, INS, MAOB, HBB, TXNRD2, GPX7, IGF1, ΕΡΗX1, COMT, and TXN2. Utilizing these meaningful genes, GSVA score (OS-Enrichment) was calculated by GSVA. An increase in the GSVA score was associated with elevated levels of SRC, TP53, TRMT10C, C1QBP, MRPS25, GTPBP3, and PDHA1, while levels of ΕΡΗX1, IGF1, TXN2, and TXNRD2 decreased (Fig 2B). An correlation analysis between GSVA score and all genes identified 58 genes with a correlation coefficient > 0.35 and *P* < 0.05. These related genes were visualized using heat maps (Fig 2C), demonstrating increased gene expression with rising GSVA score. Survival analysis indicated that patients in the high OS-Enrichment group exhibited a shorter survival rate compared to those in the Low OS- Enrichment (*P* < 0.001) (Fig 2D). Finally, 15 important genes were identified through univariate analysis (*P* < 0.05) (Fig 2E).

### Construction of oxidative stress-related gene risk model

**Fig 3:**
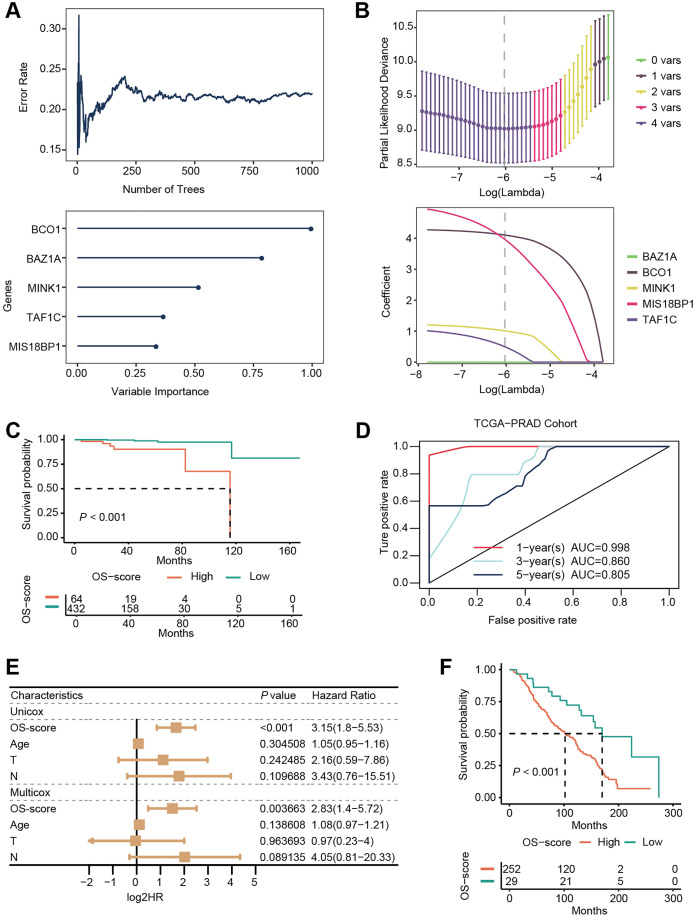
Construction of risk score. **(A)** Survival random forest analysis screening. **(B)** The genes were screened via LASSO analysis, and the lasso model was constructed. **(C)** Survival analysis in the TCGA-PRAD dataset. **(D)** ROC curve. **(E)** Univariate and multivariate regression analysis of prognostic features. **(F)** Survival analysis in the GSE16560 validation cohort.

Then, 15 important genes were subjected to further screening, resulting in the identification of 5 key genes (BCO1, BAZ1A, MINK1, TAF1C, and MIS18BP1) via survival random forest analysis (Fig 3A). LASSO analysis was performed on key genes (Fig 3B), and 4 genes (BCO1, MINK1, TAF1C, and MIS18BP1) were obtained for the construction of risk model: Risk score = 4.1028 × BCO1 + 1.0111 × MINK1 + 0.4966 × TAF1C + 3.9616 × MIS18BP1. The Kaplan-Meier method was employed to analyze survival rate between high- and low-OS-score groups, revealing that patients in the high-OS-score group experienced shorter survival times compared to those in the low- (*P* < 0.001) (Fig 3C). The ROC curve effectively identified the OS-score, with detailed AUC values of 0.998 at 1 year, 0.860 at 3 years, and 0.805 at 5 years (Fig 3D). Both univariate (*P* < 0.001) and multivariate (*P* = 0.003663) analyses demonstrated that the OS-score serves as an independent prognostic factor in PCa (Fig 3E). Utilizing the GSE16560 dataset, survival analysis indicated that patients in the low-OS-score group exhibited higher productivity compared to those in the high- (Fig 3F). These findings suggest that the OS-score significantly influences the prognosis of patients with PCa.

**Fig 4:**
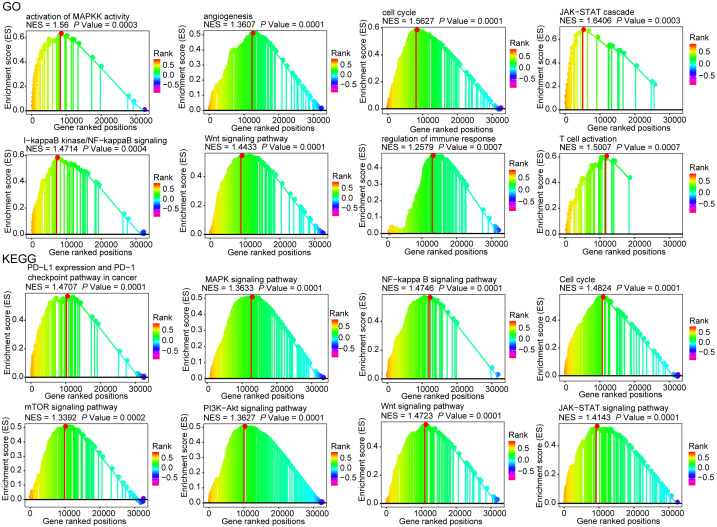
Functional enrichment analysis. GO and KEGG analysis were performed via GSEA.

### Functional enrichment analysis

OS-score related functional pathways were further analyzed by GSEA (Fig 4). In Gene Ontology (GO) enrichment and Kyoto Encyclopedia of Genes and Genomes (KEGG) pathway analysis, Cell cycle (GO: NES = 1.5627, *P* = 0.0001; KEGG: NES = 1.4824, *P* = 0.0001), NF−kappa B signaling pathway (GO: NES = 1.4714, *P* = 0.0004; KEGG: NES = 1.4746, *P* = 0.0001), and Wnt signaling pathway (GO: NES = 1.4433, *P* = 0.0001; KEGG: NES = 1.4723, *P* = 0.0001) were positively correlated with oxidative stress.

### Immune cell infiltration and immune checkpoint analysis

**Fig 5:**
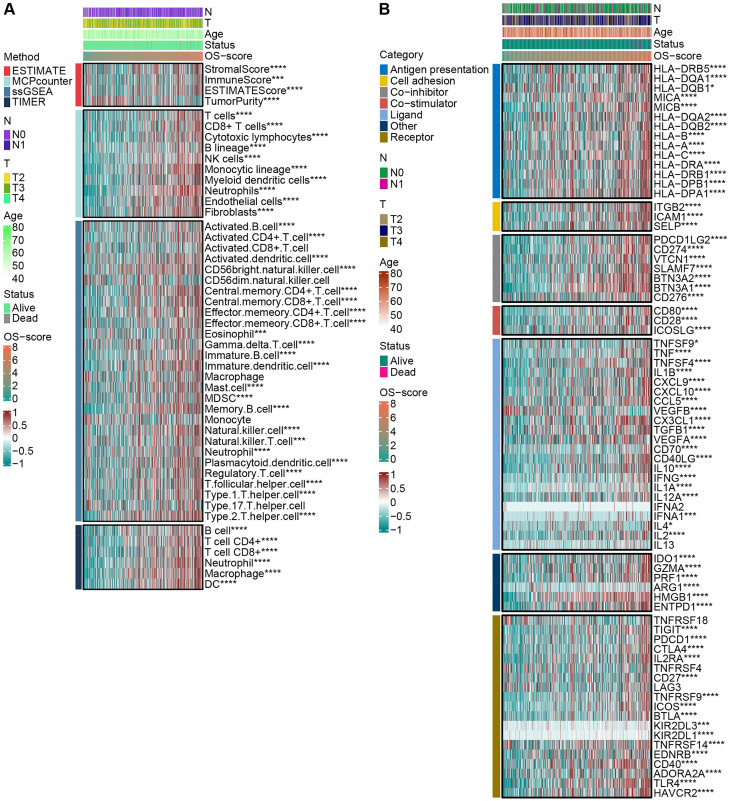
Analysis of immune cell infiltration and immune checkpoint. Immune cell infiltration **(A)** and immune checkpoint **(B)** analysis were performed.

Next, the infiltration of immune cells was evaluated using ESTIMATE, MCPCounter, ssGSEA, and TIMER (Fig 5A). The infiltration analysis revealed that various immune cell types, including Neutrophils, NK cells, CD8 + T cells, T cells, activated CD4 + T cells, macrophages, and B cells, exhibited a positive correlation with the OS-score. Further analysis was conducted to examine the correlation between OS-score and multiple immune checkpoints (including Antigen presentation, Cell adhesion, Co-inhibitor, Co-stimulator, and Ligand) (Fig 5B). The OS-score demonstrated a positive correlation with MICB, ICAM1, IL2, IL10, CXCL10, CXCL9, IL1B, and TLR4, while a negative correlation was observed with VEGFB.

### Immunotherapy analysis

**Fig 6:**
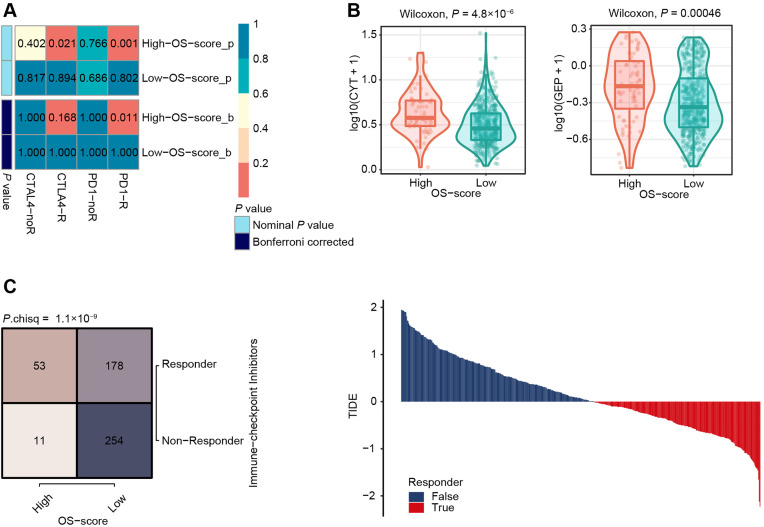
Immunotherapy analysis. **(A)** Similarity of gene expression profiles between OS-score and anti-CTAL-4 or anti-PD-1 responders was analyzed by subclass mapping. **(B)** Immune cell infiltration. **(C)** Immune checkpoint inhibitor treatment response was predicted in the high-/low-OS-score groups.

Subclass mapping analysis was employed to predict the efficacy of immunotherapy, specifically CTAL-4 and PD-1 inhibitors, between groups with high- and low-OS-score. As illustrated in Fig 6A, PCa patients in the high-OS-score group showed greater efficacy in response to anti-PD-1 treatment (Nominal *P* = 0.001, Bonferroni corrected *P* = 0.011). The cytolytic score (CYT) score was calculated to evaluate the immune effector activity [43]. T cell infiltration was assessed using T cell-inflamed gene expression profiling (GEP) [44]. The CYT score (*P* = 4.8 × 10^−6^) and GEP score (*P* = 0.00046) were significantly elevated in the high-OS-score group compared to the low- (Fig 6B). Furthermore, a significant difference was observed in the response to immune checkpoint inhibitor treatment between the high- and low-OS-score groups (*P* = 1.1 × 10^−9^), with a higher proportion of responders in the high-OS-score group compared to non-responders (Fig 6C). Conversely, the low-OS-score group exhibited the opposite trend.

### Mutation analysis and CNV analysis

**Fig 7:**
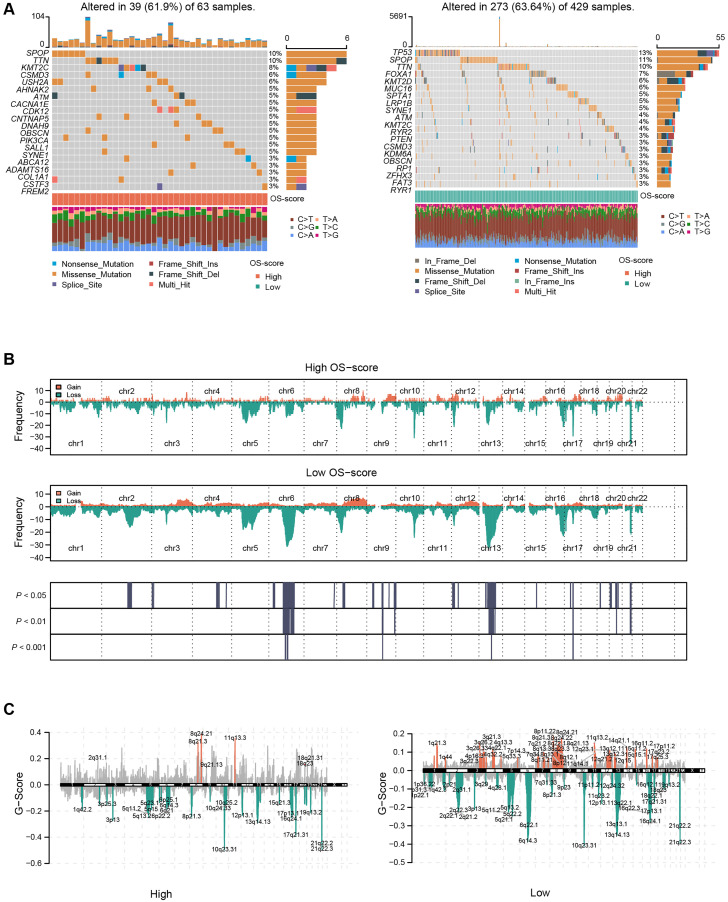
Mutation analysis and CNV analysis. **(A)** The top20 mutated genes were visualized by Oncoplot. The mutation types and the percentage (%) of patients were shown on the graph. **(B)** CNV frequence was visualized in the high-/low-OS-score groups. **(C)** The genome plot was used for CNV.

Mutation analysis revealed distinct differences in the top20 mutant genes between high- and low- groups (Fig 7A). Notably, TP53 was significantly mutated in 13% of patients in the low-OS-score group, whereas it was absent from the top 20 mutated genes in the high-OS-score group. Both SPOP (high: 10%, low: 11%) and TTN (high: 10%, low: 10%) exhibited notable mutations in the high- and low-OS-score groups. Missense mutations were the predominant type of gene mutation observed in patients across both OS-score categories. CNV analysis showed the CNV of high- and low-OS-score groups in chr6 and chr13 was significantly different (Fig 7B and 7C).

### Verification of MINK1 *in vitro*

**Fig 8:**
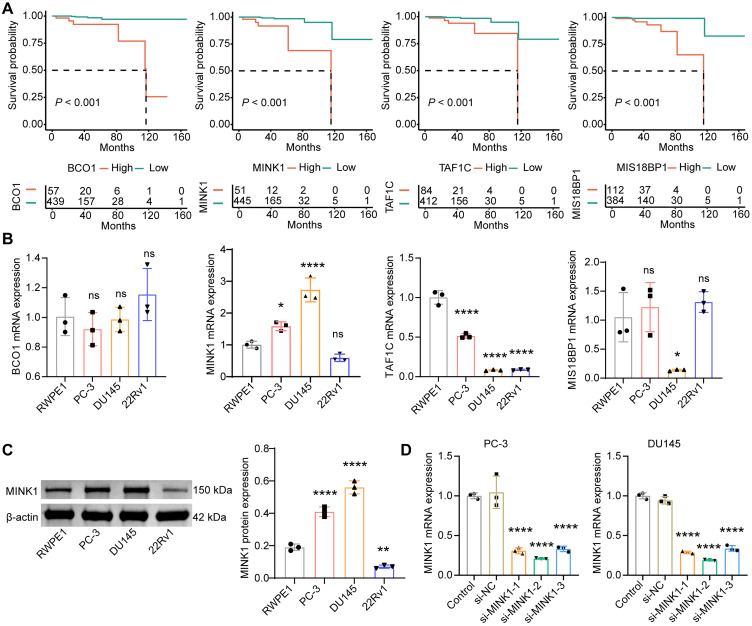
Verify the expression of MINK1. **(A)** Survival analysis of BCO1, MINK1, TAF1C, and MIS18BP1. **(B)** In the RWPE1, PC-3, DU145, and 22Rv1 cells, levels of BCO1, MINK1, TAF1C, and MIS18BP1were detected. **(C)** The protein level of MINK1. **(D)** Screening of effective silencing targets for MINK1. * *P* < 0.05, *** *P* < 0.001, and ****P < 0.0001 vs. si-NC, one-way ANOVA.

Survival analyses of these 4 genes (BCO1, MINK1, TAF1C, and MIS18BP1) are presented in Fig 8A. High levels of BCO1, MINK1, TAF1C, and MIS18BP1 were associated with a poor prognosis in patients. Further, levels of BCO1, MINK1, TAF1C, and MIS18BP1 were verified in the RWPE1, PC-3, DU145, and 22Rv1 cell lines (Fig 8B). Notably, MINK1 exhibited elevated expression in PC-3 and DU145 cells compared to RWPE1 cells. BCO1 expression did not significantly differ across RWPE1, PC-3, DU145, and 22Rv1 cells. Both TAF1C and MIS18BP1 were expressed at low levels in PCa cell lines (PC-3, DU145, and 22Rv1). The expression patterns of TAF1C and MIS18BP1 were inconsistent with the survival analysis results, potentially due to variations in sample sources and the inherent complexity of tissues and cells. Considering that MINK1 is regulated by ROS [45]. Therefore, we chose MINK1 for further analysis. Compared with RWPE1 cells, MINK1 protein levels were significantly increased in PC-3 and DU145 cells (Fig 8C). However, in 22Rv1 cells, MINK1 levels were reduced, which contradicts the prediction that MINK1 is a high-risk gene in PCa. Consequently, efficient knockdown sites for MINK1 were further screened in PC-3 and DU145 cells, and MINK1 siRNA-2 showed the best silencing effect (Fig 8D). MINK1 siRNA-2 was subsequently employed in further experiments.

### MINK1 affects the proliferation, migration, and invasion activity of PCa cells

**Fig 9:**
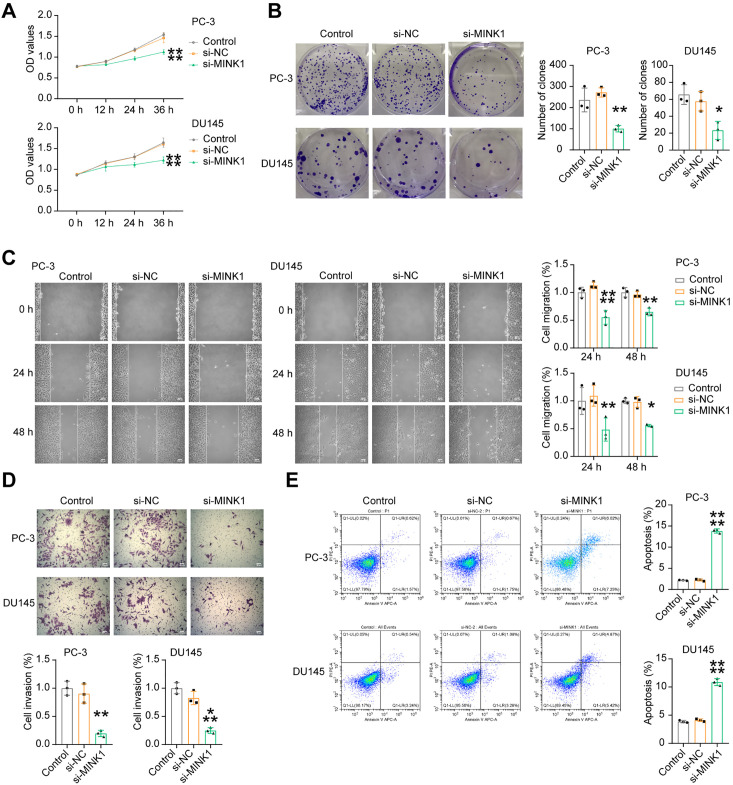
Regulation of MINK1 affects the proliferation, migration, and invasion. **(A)** After MINK1 intervention, CCK-8 detected the proliferative ability at 0 h, 12 h, 24 h, and 36 h**. (B)** Plate cloning was applied to analyze cell proliferation. **(C)** Scratch analysis was performed to measure migration activity after MINK1 silencing for 24 h and 48 h**. (D)** Transwell was adopted to detect the cell invasion levels. **(E)** Apoptosis rates were measured by FCM. * *P* < 0.05, ** *P* < 0.01, *** *P* < 0.001, and **** *P* < 0.0001, vs. si-NC, one-way ANOVA.

The suppression of MINK1 expression resulted in a significant reduction in the proliferative capacity of PC-3 and DU145 cell lines (Fig 9A). Clonogenic assays demonstrated a marked decrease in proliferation within the si-MINK1 group compared to the si-NC (Fig 9B). Furthermore, the migratory and invasive capabilities of the cells were inhibited in the si-MINK1 (Fig 9C and 9D). Apoptosis rates of PC-3 and DU145 increased after the intervention of MINK1 siRNA (Fig 9E). Both DU145 and PC-3 cells became less migratory and less invasive after silencing MINK1.

### MINK1 regulates cell cycle and NF-κB signaling pathway

**Fig 10:**
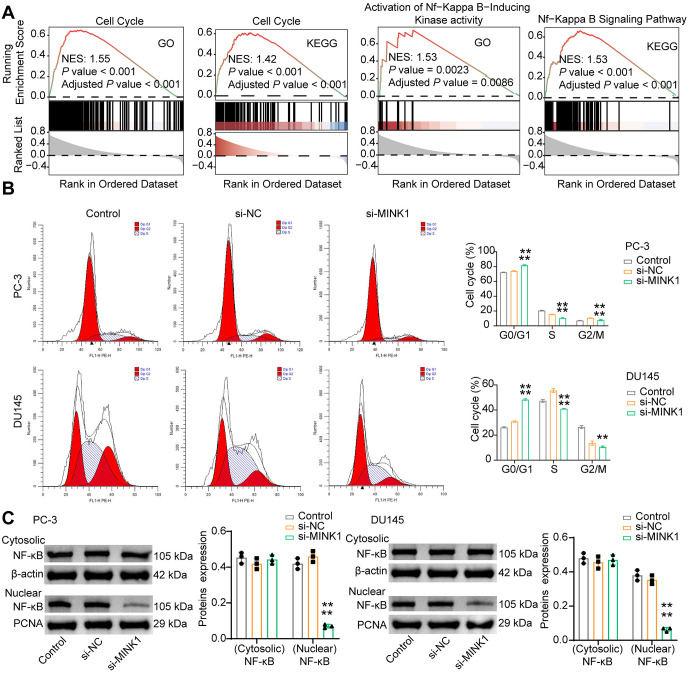
MINK1 regulates cell cycle and NF-κB signaling pathway in PCa. **(A)** Functional enrichment analysis related to MINK1. **(B)** Flow cytometry was conducted to analyze cell cycle. **(C)** NF-κB (nuclear and cytoplasm) was detected by Western blot. ** *P* < 0.01 and **** *P* < 0.0001, vs. si-NC, two-way ANOVA.

Prior functional analyses results (Fig 4) suggested an association between the cell cycle, NF-κB signaling pathway and OS-score. Further functional enrichment analysis revealed that MINK1 positively regulates the cell cycle (GO: NES = 1.55, *P* < 0.001; KEGG: NES = 1.42, *P* < 0.001), activation of NF-κB-inducing kinase activity (GO: NES = 1.53, *P* = 0.0023), and NF-κB signaling pathway (KEGG: NES = 1.53, *P* < 0.001) (Fig 10A). Subsequently, under the condition of MINK1 siRNA treatment, the cell cycle levels and the nuclear translocation of NF-κB were identified. The proportion of G0/G1 phase in the si-MINK1 group was higher than in the si-NC, while the proportion of S phase was suppressed (Fig 10B). These showed that silencing MINK1 could induce G0/G1 phase arrest in PC-3 and DU145 cells. Next, we identified the levels of NF-κB in the nucleus and cytoplasm. NF-κB (nuclear) levels were significantly decreased in PC-3 and DU145 cells (Fig 10C). The decrease of cytosolic NF-κB was not obvious.

## Discussion

Oxidative stress is crucial for maintaining cellular homeostasis, and its regulation can mitigate diseases progression [46,47]. The study investigated the impact of oxidative stress-related genes on the development of PCa. Combined with the clinical characteristics of patients with the TCGA-PRAD and GSE16560 data, the prognosis of PCa patients was predicted.

Oxidative stress genes were obtained from the GeneCards, and 22 significant genes were identified through univariate analysis, including NFS1, PDHA1, C1QBP and SRC. Previous research has demonstrated that NFS1 defects can stimulate changes the intracellular ROS levels in cancer cells [48]. In the hypoxia/reoxygenation-induced cardiac muscle cells injury, PDHA1 are involved in the regulation of energy metabolism and oxidative stress [49]. Additionally, C1QBP is implicated in the regulation of intracellular oxidative stress and mitochondrial function [50,51]. Cellular Src (c-Src) in hypertension is closely related to oxidative stress, and activation of c-Src can promote the overproduction of ROS [52]. Collectively, these studies corroborate the reliability of our analytical findings. Subsequently, BCO1, MINK1, TAF1C, and MIS18BP1 were screened to construct the OS-score through correlation analysis, survival random forest and Lasso analysis. The survival rate of patients in the high-OS-score group was lower than that in the low-. Univariate (*P* < 0.001) and multivariate (*P* = 0.003663) analyses demonstrated that the OS-score was an independent prognostic factor for PCa patients. These findings suggest that oxidative stress is a promising classification criterion in the diagnosis and prognosis prediction of prostate cancer patients.

Gene mutation and CNV were predicted and found to be markedly different between the high-/low- groups. PCa is a typical heterogeneous cancer. Results indicate that oxidative stress can affect mutation and CNV in PCa to a certain extent. Additionally, the infiltration of immune cells was also obviously correlated with the OS-score. Our data analysis shown that CD8 + T cells, CD4 + T cells, NK cells, and macrophages were positively correlated with OS score. Previous studies have shown that oxidative stress can guide the adaptive metabolism of CD8 + T cells in the tumor microenvironment (TAM) [53,54]. Genes involved in oxidative stress are enriched in CD4 + T cells of PCa [55]. Oxidative stress regulatory factors can drive the activation and expansion of CD4 + T cells by regulating glucose and glutamine metabolism [56]. The presence of hydrogen peroxide within the tumor stroma influences the functionality of NK cells [57]. NK cells exhibit greater sensitivity to hydrogen peroxide compared to T cells or B cells [57]. Hypoxic conditions promote the polarization of M2-type tumor macrophages in the TAM, and inhibition of oxidative stress can induce repolarize of M2-TAMs [58]. Furthermore, macrophage-derived inflammatory and immune processes are also associated with oxidative stress [59,60]. Oxidative stress plays a role in the biological processes of immune cells, suggesting that it may impact the PCa development by influencing the function and phenotypic regulation of these cells. MINK1, a critical gene involved in oxidative stress, can affect the progression of inflammatory disorders by modulating the inflammatory activation in macrophages [45]. Based on the aforementioned analysis, oxidative stress-related genes may influence the development of PCa by regulating the function and phenotypic changes of immune cells. Immune cells may be involved in the PCa treatment. Meanwhile, our data indicate that patients with high-OS-score responded more effective in anti-PD-1 therapy. The proportion of responders in the high-OS-score group was greater than that of the non-responders in the context of immune checkpoint inhibitor treatment. These findings support our hypothesis that immunotherapy may yield favorable outcomes in patients with high-OS-score. However, due to financial and temporal limitations, this will be our subsequent objective. In the future, we will further analyze the role of oxidative stress in PCa immunotherapy through *in vivo* and *in vitro* experiments.

The ROS stimulation can promote the activation of MINK1 [45], which is closely related to oxidative stress. Therefore, MINK1 was selected for further analysis. MINK1 was highly expressed in PC-3 and DU145 cells. PC-3 and DU145 cells are both androgen-independent human PCa cell lines with high metastatic potential. MINK1 is enriched in highly metastatic cells. Silencing MINK1 significantly reduced cell proliferation, migration, and invasion, suggesting that MINK1 might be involved in the metastasis process of PCa. MINK1 can promote cancer cell migration by forming a PRICKLE1‑MINK1‑RICTOR complex with PRICKLE1 and RICTOR [61]. This provides further evidence to support our viewpoint. On the other hand, combined with functional enrichment analysis, our study identified the involvement of the cell cycle and NF-κB signaling pathways in PC-3 and DU145 cell lines. Inhibition of MINK1 resulted in cell arrest at the G1/S phase in these cells. MINK1, a member of germinal center kinase family, interacts directly with STRN4 to regulate cell division [62]. Consistent with our findings, inhibition of MINK1 has been shown to induce G1/S phase arrest in colon cancer cells [63]. Furthermore, silencing MINK1 inhibited the nuclear translocation of NF-κB, indicating that MINK1 regulates NF-κB activation. Increased oxidative stress is frequently associated with NF-κB activation [64,65], and inhibition of NF-κB can enhance the antioxidant capacity of cells [66]. Our data suggest that MINK1 may modulate oxidative stress levels in PCa cells via the NF-κB signaling pathway. Meanwhile, inhibition of the activation of NF-κB can induce apoptosis of cancer cells [67]. Therefore, MINK1 may influence cell proliferation via regulating the cell cycle and NF-κB signaling pathway.

### Limitations

Several limitations need to be considered when interpreting the data presented in this study. Firstly, although oxidative stress-related gene signatures have been associated with immune characteristics and the response to immunotherapy in the development of PCa, the changes in gene expression have not been validated and confirmed *in vivo*, leaving their role uncertain. Furthermore, the function of MINK1, as a marker of oxidative stress-related gene signature, in the development of PCa has not been further verified *in vivo*, and its downstream regulatory pathways in PCa remain unexplored.

## Conclusion

Oxidative stress was an independent prognostic factor for PCa by analyzing both TCGA-PRAD and GSE16560 cohort. Our experimental data suggest that the key oxidative stress gene MINK1 may regulate the proliferation, migration, and invasion activity of PCa cells (PC-3 and DU145), potentially through modulation of the cell cycle and NF-κB signaling pathways. In conclusion, oxidative stress may serve as a predictive marker for the diagnosis and prognosis of PCa patients. This study provides a scientific basis for future PCa treatment strategies.

## Supporting information

S1 File Collection of raw data. (XLSX)

S2 File Western blots. (PDF)
