# Supplementary material for: Identification of the oxidation stress-related gene signatures and functional verification of MINK1 in prostate cancer cells
Source: PLoS One. 2026 Jul 8;21(7):e0350334. doi: 10.1371/journal.pone.0350334 (PMC13345288; doi:10.1371/journal.pone.0350334)

Fig. 8B

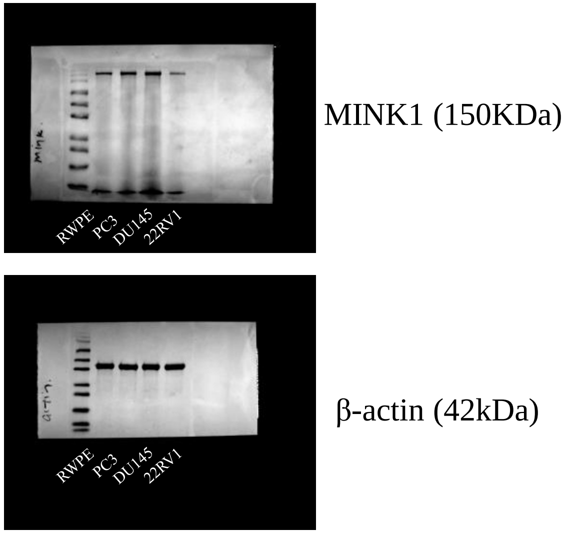

Fig. 10B (DU145)

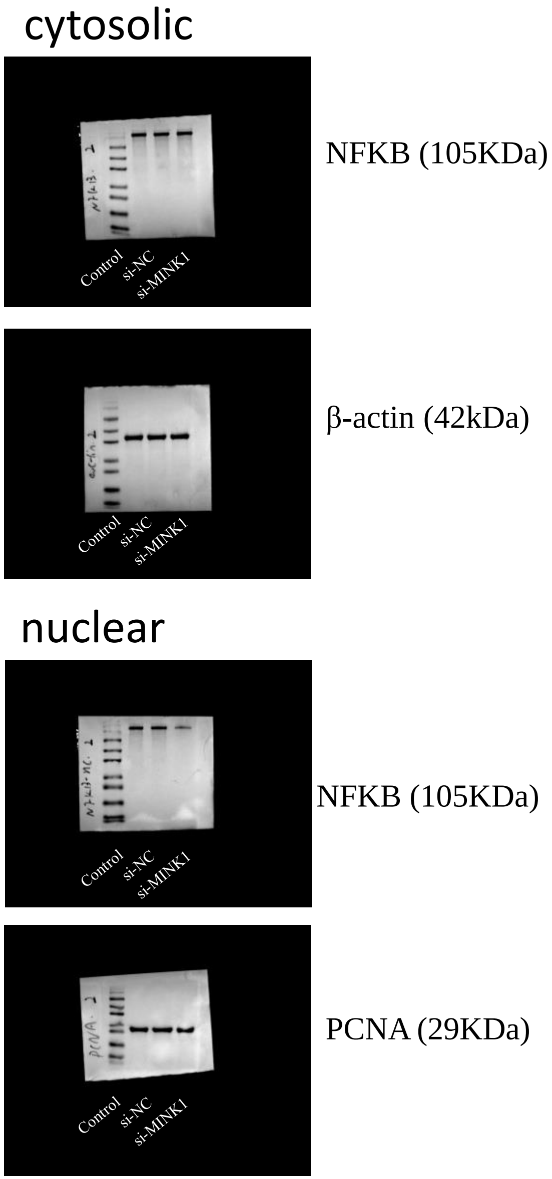

Fig. 10B (PC-3)

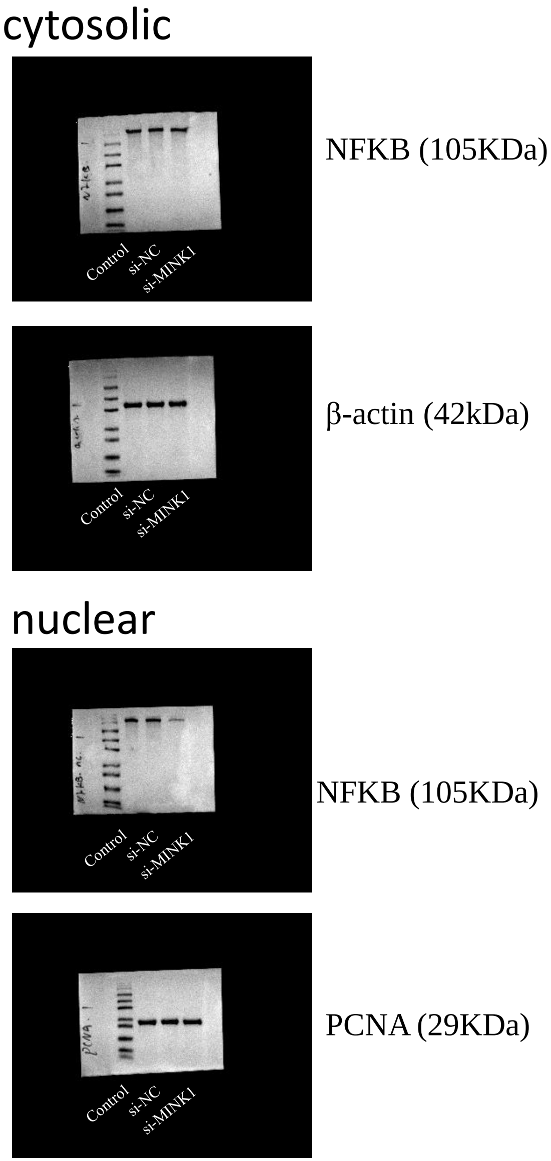

Supplement: S2 File — (PDF) [file pone.0350334.s002.pdf]
